# Mesenchymal Transition and PDGFRA Amplification/Mutation Are Key Distinct Oncogenic Events in Pediatric Diffuse Intrinsic Pontine Gliomas

**DOI:** 10.1371/journal.pone.0030313

**Published:** 2012-02-28

**Authors:** Stephanie Puget, Cathy Philippe, Dorine A. Bax, Bastien Job, Pascale Varlet, Marie-Pierre Junier, Felipe Andreiuolo, Dina Carvalho, Ricardo Reis, Lea Guerrini-Rousseau, Thomas Roujeau, Philippe Dessen, Catherine Richon, Vladimir Lazar, Gwenael Le Teuff, Christian Sainte-Rose, Birgit Geoerger, Gilles Vassal, Chris Jones, Jacques Grill

**Affiliations:** 1 Department of Neurosurgery, Necker-Sick Children Hospital, University Paris V Descartes, Paris, France; 2 Unite Mixte de Recherche 8203 du Centre National de la Recherche Scientifique «Vectorology and Anticancer Therapeutics», Gustave Roussy Cancer Institute, University Paris XI, Villejuif, France; 3 Section of Pediatric Oncology, The Institute of Cancer Research/Royal Marsden Hospital, Sutton, Surrey, United Kingdom; 4 Formation de Recherche en Evolution 2939 du Centre National de la Recherche Scientifique, Integrated Research Cancer Institute in Villejuif, University Paris XI, Villejuif, France; 5 Team Glial Plasticity, Unite Mixte de Recherche 894 de l'Institut National de la Santé et de la Recherche Medicale and Department of Neuropathology, Sainte-Anne Hospital, University Paris V Descartes, Paris, France; 6 Life and Health Sciences Research Institute, University Do Minho, Braga, Portugal; 7 Center for Neuroscience and Cell Biology, University of Coimbra, Coimbra, Portugal; 8 Functional Genomics Unit, Gustave Roussy Cancer Institute, University Paris XI, Villejuif, France; 9 Department of Biostatistics and Epidemiology, Gustave Roussy Cancer Institute, University Paris XI, Villejuif, France; 10 Department of Pediatric and Adolescent Oncology, Gustave Roussy Cancer Institute, University Paris XI, Villejuif, France; The University of Chicago, United States of America

## Abstract

Diffuse intrinsic pontine glioma (DIPG) is one of the most frequent malignant pediatric brain tumor and its prognosis is universaly fatal. No significant improvement has been made in last thirty years over the standard treatment with radiotherapy. To address the paucity of understanding of DIPGs, we have carried out integrated molecular profiling of a large series of samples obtained with stereotactic biopsy at diagnosis. While chromosomal imbalances did not distinguish DIPG and supratentorial tumors on CGHarrays, gene expression profiling revealed clear differences between them, with brainstem gliomas resembling midline/thalamic tumours, indicating a closely-related origin. Two distinct subgroups of DIPG were identified. The first subgroup displayed mesenchymal and pro-angiogenic characteristics, with stem cell markers enrichment consistent with the possibility to grow tumor stem cells from these biopsies. The other subgroup displayed oligodendroglial features, and appeared largely driven by PDGFRA, in particular through amplification and/or novel missense mutations in the extracellular domain. Patients in this later group had a significantly worse outcome with an hazard ratio for early deaths, ie before 10 months, 8 fold greater that the ones in the other subgroup (p = 0.041, Cox regression model). The worse outcome of patients with the oligodendroglial type of tumors was confirmed on a series of 55 paraffin-embedded biopsy samples at diagnosis (median OS of 7.73 versus 12.37 months, p = 0.045, log-rank test). Two distinct transcriptional subclasses of DIPG with specific genomic alterations can be defined at diagnosis by oligodendroglial differentiation or mesenchymal transition, respectively. Classifying these tumors by signal transduction pathway activation and by mutation in pathway member genes may be particularily valuable for the development of targeted therapies.

## Introduction

Brain tumors are the leading cause of cancer-related morbidity and mortality in children and adolescents, malignant gliomas carrying the worst prognosis among them [1]. Malignant gliomas that diffusely infiltrate the brainstem appear almost exclusively during childhood and adolescence and have a relatively homogenous presentation and dismal prognosis. DIPG represent the biggest therapeutic challenge in pediatric neuro-oncology with a median survival of 9 months despite collaborative efforts to improve treatment [2]. The vast majority of children succumb to their disease within 2 years of diagnosis. These tumors are unresectable and radiotherapy is the only treatment offering a significant but transient improvement. The addition of chemotherapy has not shown any benefit over the use of irradiation only [2], [3]. The development of targeted therapies for DIPG has been hampered by the lack of knowledge of the biology of this devastating disease. Trials have been implemented so far based on the assumption that biologic properties of these brainstem gliomas of children are identical to cerebral high-grade gliomas of adults [4], [5]. Recent data suggest however that pediatric high-grade gliomas differ from their adult counterparts [6]–[9], and that there may be biological distinctions between childhood gliomas presenting in the brainstem compared with supratentorial ones [10].

Comprehensive genomic studies of a substantial number of DIPG at diagnosis have not yet been undertaken due to the lack of available tumor material. Indeed, diagnosis is usually based on the association of specific neurological signs, short clinical history with a typical radiological appearance on MRI [11]. A biopsy is not needed for diagnosis in most of the cases [12], [13]. In addition, most of these lesions are infiltrating and grading according to the WHO classification does not correlate with outcome. Accordingly and despite the reported safety of the procedure [14], most of the neurosurgical teams limit the use of stereotactic biopsies to the lesions with unusual clinical or radiological characteristics. Therefore, only very limited data on true DIPG is available in the literature and confounded by the inclusion of autopsy – ie post-radiotherapy - cases [10], [15]–[18].

Recently, our group started to use stereotactic biopsies of DIPG to obtain both pathological confirmation and immunohistochemical assessment of some specific biomarkers before the inclusion of patients in trials of targeted agents [19]–[21]. In this study, we sought to comprehensively define genetic alterations in DIPG at diagnosis by performing genome-wide array CGH and gene expression studies from frozen samples obtained by stereotactic biopsies. This study is the first to comprehensively define the biological alterations of DIPG at diagnosis, allowing the discovery of novel therapeutic targets directed specifically at these poor prognosis brain neoplasms.

## Results

### DIPG Biopsy Material

Over the 5 years of the study, 61 patients underwent stereotactic biopsies taking from one to eight tumor samples (median 3) in the Neurosurgery Department of Necker Sick Children's Hospital in Paris. In most instances, one or two biopsies were used for histological diagnosis and immunohistochemistry (Figure S1A). The remaining biopsies were snap-frozen with cytological control smears directly in the operating room, and nucleic acids extracted from representative samples. A median of 3.325 microg of DNA (range 0.805 to 21.5 microg) and 2.332 microg of RNA (range 0.048 to 15.84 microg) could be extracted from the biopsies, resulting in a total of 32 and 23 patients with sufficient quality and quantity of DNA and RNA, respectively, for microarray analyses without any amplification step.

A second set of surgical samples from pediatric non-brainstem high-grade gliomas of various histologies with arrayCGH (n = 34) and gene expression (n = 53) data acquired simultaneously on the same platform was used for comparative studies. Age distribution at diagnosis was similar in DIPG and in HGG.

### DIPG Differ from Supratentorial High-grade Gliomas but Co-segregate with a Subgroup of Midline/thalamic Tumors

We first performed array CGH on the 32 frozen biopsies of newly diagnosed DIPG, and compared the high resolution DNA copy number profiles with a series of 34 pediatric supratentorial high grade gliomas. Unsupervised hierarchical clustering of the DIPG samples using the Euclidian distance defined two distinct subgroups, the first characterized by gain of chromosome 1q, and the second by numerous copy number losses and structural rearrangements (Figure S1B). There were no associations between array CGH subgroup and survival, age at onset, duration of symptoms before diagnosis, radiological characteristics or WHO grade according to the 2007 revision.

Amplifications at specific loci were detected by CGHarray for the oncogenes *HRAS* (5), *PDGFRA* (4), *PDGFB* (2), *CAV1/2* (2), *PTPRN2* (2), *KDM5A* (2), *ETS1* (1), *MYCN* (1), *WNT2* (1), *RAB31* (1). Deletions were detected for *PTEN* (1), *CDKN2A/B* (1) and *FAS* (1). The oncogene *H-RAS* was gained or amplified in 7/32 (22%) and the *TP53* tumor suppressor gene was lost in 7/32 (22%) of cases. Loss of *TP53* locus was the only single chromosomal imbalance associated with a poorer outcome (p = 0.01, log-rank test) (Figure S1C). On immunohistochemistry, p53 overexpression was seen in 15/27 (55%) cases. A comprehensive list of minimal common regions of imbalances with a frequency superior to 15% is provided in Table S1.

**Figure 1:**
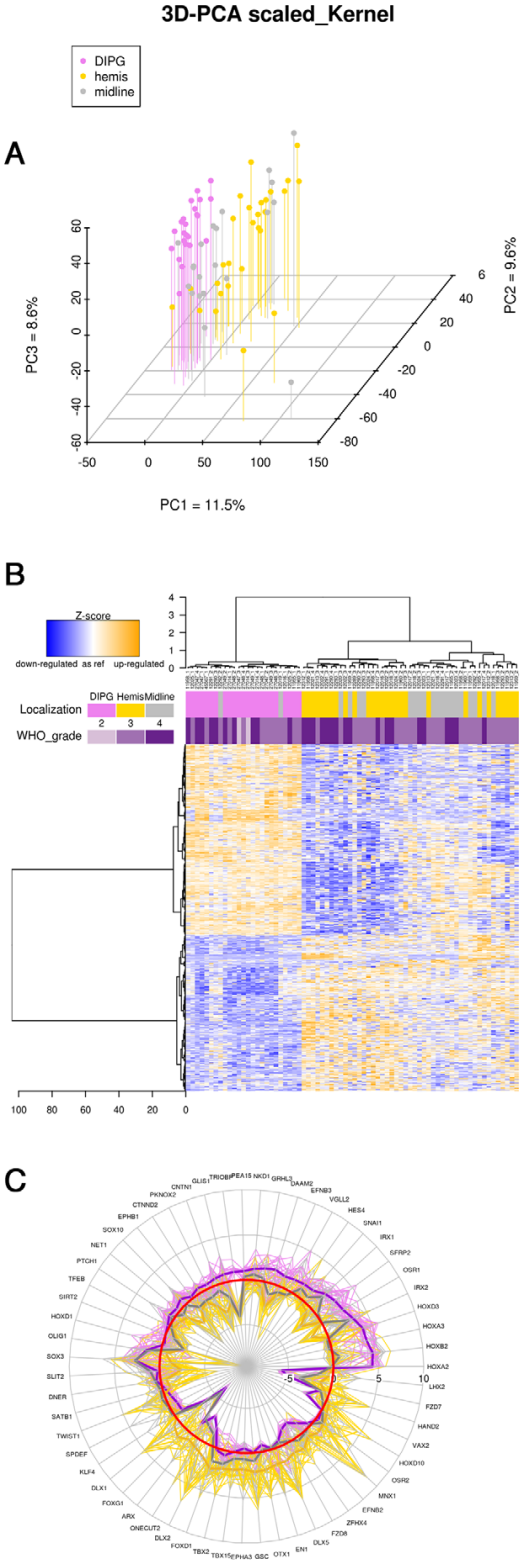
DIPG are different from supratentorial high-grade gliomas in children. Hemispheric, midline/thalamic tumors and DIPG are represented in gold, grey and violet respectively. Panel A: Gene expression of 23 DIPG and 49 supratentorial HGG were compared using a Principal Component Analysis on all 15231 quality control passing probes. Tumors are displayed according to their coordinates on the three first principal components, which describe 29.7% of the variance. Panel B: Heatmap of the 712 most differentially expressed genes between DIPG, midline and hemispheric tumors, selected using the moderated t-test of limma package of Bioconductor. Panel C: Radial plot of the expression of transcription factors and neurogenesis regulators according to the three tumor location, in log2 ratios related to normal brain stem. The zero red line represent the expression level of normal adult brainstem.

It was not possible to clearly delineate DIPG and supratentorial tumors on the basis of the copy number profiles, as exemplified by an unsupervised principal component analysis (PCA) generated using all 42332 quality control passing probes (Figure S1D). By contrast, a similar PCA analysis of gene expression profiling using all 15231 quality control passing gene probes demonstrated the clustering of the DIPG samples distinct from the majority of supratentorial high-grade gliomas, with the exception of some midline (thalamic) tumors (Figure 1A).

Supervised analysis using the 76 samples (23 DIPG and 53 HGG) was used to identify the genes most closely associated with pediatric high-grade gliomas arising in the brainstem *versus* supratentorially, and resulted in an expression signature comprising 712 genes (p<0.005, Pearson correlation, Ward procedure) which could distinguish tumours based on location independent of WHO grade (Table S2). The corresponding heatmap showed that the GE profiles of midline tumors clustered in some cases with the ones of DIPG (Figure 1B). Figure 1C shows the distribution of the expression for transcription factors and neurogenesis regulators according to the three different locations. DIPG and supratentorial tumors could be distinguished by a different pattern of expression of specific homeobox and HLH genes. When analysing the expression levels of the major regulators of brainstem embryogenesis described in the literature, we observed a significant upregulation of *GAL3ST1*, *MAFB*, *OLIG2* and *HOXA2,3* and *4* in DIPG compared to supratentorial tumors (Figure S1E).

### DIPG Comprise two Biological Subgroups with Distinct Survival and Pathological Characteristics

**Figure 2:**
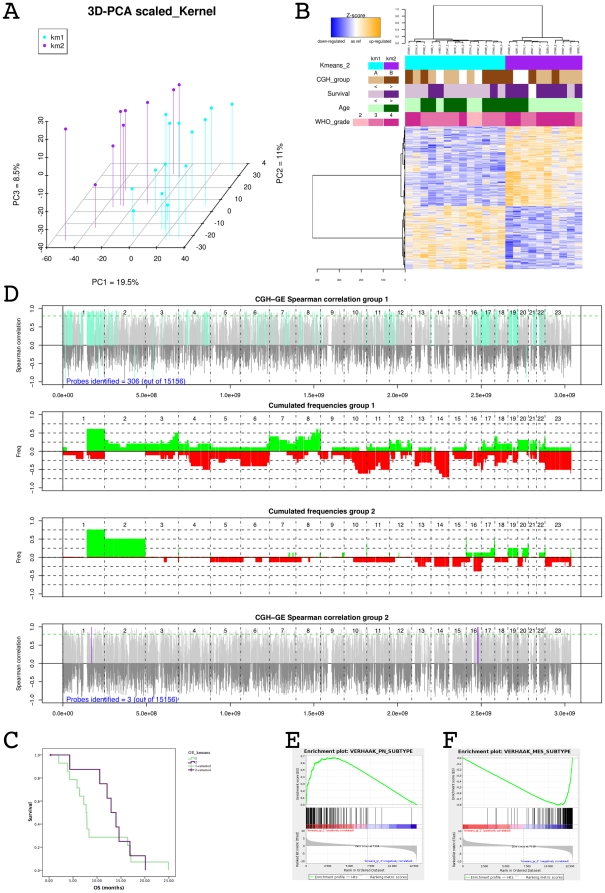
DIPG are divided into two groups with different gene expression signatures. Gene expression levels of 23 DIPG were analysed using a unsupervised procedure. Panel A: K-means algorithm followed by a model selection procedure using BIC defined two separated groups of DIPG that can be also clearly seen with a PCA on all probes that passed the quality control. Panel B: Heatmap of the 643 most differentially expressed genes between the two groups of DIPG, selected using the moderated t-test of limma package of Bioconductor. Panel C: Overall survival curves of the two groups of DIPG defining a group of patients who died early (70% of cases before the median survival time of 10.6 months, light green curve) and a group of patients who died later (90% of cases after the median survival time of 10.6 months, purple curve), (p = 0.004, chi-square test). Panel D: Integrated genomic analysis using DR-Integrator (R package) showing the correlation between probes of copy number and gene expression mapped on the same genomic coordinates (Refseq HG19) of a gene. In the upper panel (resp. the lower panel), colored vertical lines (cyan for group 1, purple for group 2) show probes for which copy number and expression were significantly correlated. The two panels in the middle show the CNA frequencies for the group 1 (resp. for the group 2). Most of the correlations between GE and CGH were found in group 1. Gene set enrichment analysis (GSEA) plot comparing group 1 GE profile to the signatures described for adult type gliomas. Group 1 gene expression profiles were enriched for proneural genes (Panel E) while group A gene expression profiles were enriched for mesenchymal genes (Panel F).

The unsupervised k-means algorithm was used to discover subgroups of DIPG based on their gene expression profiles. The most optimal Bayesian Information Criterion (BIC) value was obtained for the classification based on two clusters [22] (Figure S2A), as represented by the corresponding principal component analysis (Figure 2A). Supervised hierarchical clustering identified 643 genes differentially expressed between these two groups (False Discovery Rate (FDR) adjusted p-value<0.01) (Table S3 and Figure 2B). The first group had a significantly worse survival, with 70% (9/13) of children succumbing to the disease before the median overall survival time of 10.6 months (range 2 to 25 months) of the entire cohort, whilst only 10% (1/10) of the patients in the second group did so (Figure 2C). Since the risk of death was not proportional over time in the two groups, we use a Cox model with an interaction between group and time. The hazard ratio for early deaths, ie before 10 months, was 0.122 for group 2 vs group 1 (p = 0.041). Significant association of the 2 GE groups was observed neither with age nor with the array CGH classification described above.

Integrative analysis of the copy number and expression profiles using Spearman correlations demonstrated a significant influence of copy number on gene expression in group 1 tumours (306/15189 = 2% probes significantly correlated), however not for those in group 2 (3/15189 = 0.02% probes significantly correlated) (Figure 2D). These strong correlations were restricted to certain chromosomal abnormalities, in particular gain of 1q, loss of 19q, and amplification of 4q12. When considering both groups together the expression of 1460 genes (6% of the genome) was significantly correlated with their copy number; six of the twenty most correlated genes were located on chromosome 4q12 region: *CHIC2*, *SRP72*, *CLOCK*, *PPAT*, *SRD5A3* and *EXOC1* with Spearman correlation coefficient >0.9 and adjusted p<0.01 (Figure S2B).

Using gene set enrichment analysis [23], the expression profiles of the two groups were compared with the four subgroups of adult high grade gliomas recently described as proneural, neural, classical/proliferative and mesenchymal (http://tcga-data.nci.nih.goc/docs/publications/gbm_exp/) [24]. The proneural signature was highly enriched in the gene expression signature of group 1 (enrichment score = 0.66; nominal p = 0.004; FDR q = 0.089) (Figure 2E) while the mesenchymal signature was significantly associated with group 2 tumours (enrichment score = 0.8; nominal p = 0.004; FDR q = 0.007) (Figure 2F).

### Mesenchymal Transition and a Pro-angiogenic Switch Define A Subset of DIPG

**Figure 3:**
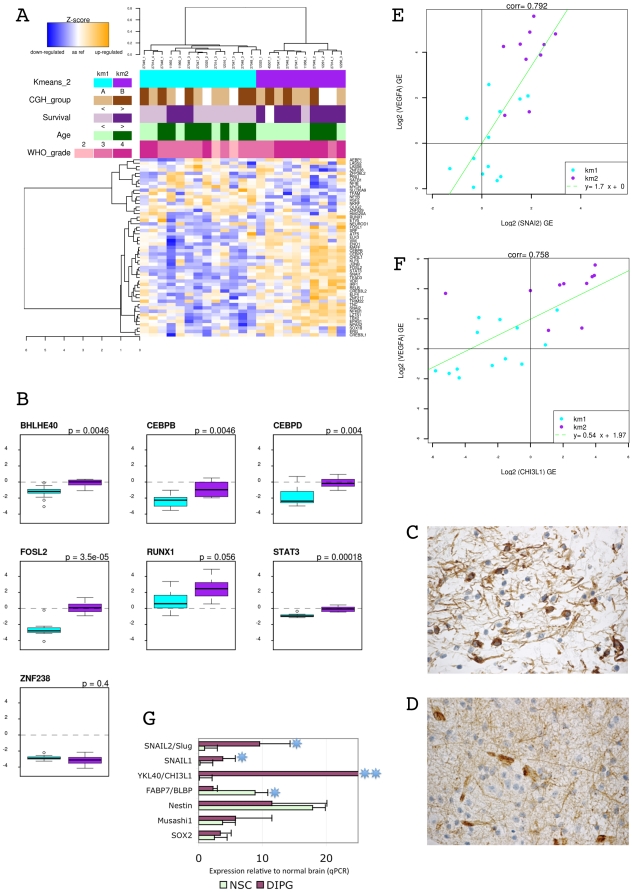
Description of the mesenchymal type of DIPG. DIPG from group 2 gene expression profile was enriched with genes involved in mesenchymal transition, angiogenesis and stem cell maintenance. Panel A: Heatmap of the transcription factors linked with mesenchymal gene expression signature (MGES) in adult glioblastomas. Biomarkers of mesenchymal phenotype (VIM, CHI3L1 and TNC) and the two master regulators of epithelio-mesenchymal transition, SNAIL1 and SNAIL2/SLUG were added to the list provided by Carro et al (Carro et al., 2010). Panel B: Boxplots comparing the 7 transcription factors driving the MGES in adult glioblastomas (Carro et al., 2010) in the two groups of DIPG (group 1 in cyan, group 2 in purple). Relative expression in log2 ratio compared to normal brainstem control is indicated. Vimentine immunohistochemistry in tumors of group 2 shows the positivity of tumors cells (Panel C) while in group 1 only vessels and reactive astrocytes were positive (Panel D). Panel E: Spearman correlation of the expression of SNAI2 and VEGFA. Group 1 tumors (cyan dots) segregate clearly from tumors of group 2 (purple dots). Panel F: Spearman correlation of the expression of CHI3L1 and VEGFA. Group 1 tumors (cyan dots) segregate clearly from tumors of group 2 (purple dots). Panel G: Gene expression of stem cell and mesenchymal markers in DIPG tumorospheres derived from primary tumors of patients in stem cell medium as previously described (Thirant et al, 2011). Quantitative RT-PCR (qPCR) were performed using normal brain cortectomy as control. The spheroids cultured from three different DIPG were compared to normal neural stem cells (NSC) grown as neurospheres in the same medium.

Since a mesenchymal gene expression signature was specifically represented in one of the two DIPG expression groups, we compared the expression of 53 transcription factors specific for this process as previously defined in adult high grade gliomas [25]. These genes were significantly upregulated in the group 2 DIPGs relative to the group 1 tumours (GSEA analysis: enrichment score 0.56, FDRq = 0.039, p nominal = 0.034), together with the master epithelial-mesenchymal transition regulators, *SNAI1* and *SNAI2/Slug* genes (Figure S3A). Expression of these genes alone was sufficient to distinguish group 1 and group 2 DIPG (Figure 3A). A subset of 7 transcription factors (*STAT3, BHLHE40, CEBPA and B, RUNX1, FOSL2 and ZNF238*) controlled most genes of the mesenchymal signature of gliomas; all but *ZNF238* were significantly upregulated in the group 2 tumours compared to the other DIPG (Figure 3B). This transcriptional module was associated with a mesenchymal phenotype with upregulation of *TNC*, *OSMR*, *VIM* and *YKL40/CHI3L1* and a more astrocytic histology (Table S3 & Figures 3A and 3C/D). Knowing that the *BRAF* V600E mutations could induce mesenchymal transition in some tumors [26] and that such mutations have been reported in a subset of pediatric glioma [27], we sequenced exon 15 of the *BRAF* gene in 20 of the DIPG irrespective of their subgroup. No mutation was detected.

This mesenchymal phenotype was coupled with a hypoxia-induced angiogenic switch. Numerous proangiogenic genes were significantly overexpressed in this subgroup of DIPG compared to the other ones, including *VEGFA*, *VWF*, *PECAM1*, *TREM1, OSMR* and *PLAU* (Table S3 and Figure S3B). There was a strong correlation between *VEGFA* and *SNAI2/Slug* expression (Figure 3E), and between *VEGFA* and *YKL40* (Figure 3F) across the entire dataset, with a clear separation of the tumors in the two groups defined by the gene expression profiling. Endothelial proliferation was present in 8/9 mesenchymal group 2 tumours vs 8/14 in group 1 (89% vs 57%, p = NS, chi square test). On the extended cohort of 54 FFPE samples where endothelial proliferation could be evaluated, there was no correlation with survival, however an inverse correlation with Olig2 immunopositivity, a core biomarker of the proneural signature was noted (p = 0.01, chi square test). This angiogenic switch was associated with the activation of the *HIF1A* pathway as shown by the higher expression of *HIF1A* in group 2 (p = 0.058, Student t-test) and by the significant overexpression compared to group 1 of 5/8 of the hypoxia-related genes whose promoter is known to be highly responsive to *HIF1A: ENO2, HK1, HK2, LDHA, P4HA2* (Table S3).

This mesenchymal profile was further associated with a significant overexpression of numerous stem cell markers, including *BMI1*, *CD34, CD44, CXCR4, LIF, DKK1, VIM* and *RUNX2*, in group 2 versus group 1 tumours (Figure S3C). Association of mesenchymal and stem cell markers was conserved in tumor cells with stem-like properties derived from three independent DIPG biopsies. These tumor stem-like cells yielded phenocopies of the original tumors in intracerebral xenografts (for complete description see [28]) and had a molecular profile as seen by qPCR similar to fetal neural stem cells with respect to stem cell markers (ie SOX2, Musashi1, Nestin and FABP7/BLBP) while overexpressing the mesenchymal markers YKL40, SNAIL1 and SNAIL2 compared to normal neural stem cells (Figure 3G). Of note, none of these tumor stem cells cultures, showed PDGFRA overexpression or amplification. The gene expression profile obtained from one of these DIPG models resembled mesenchymal subtype of DIPG as shown by unsupervised clustering using PCA (Figure S3D).

### Oligodendroglial Differentiation and PDGFRA Amplification/mutation Define the Remaining Subset of DIPG

**Figure 4:**
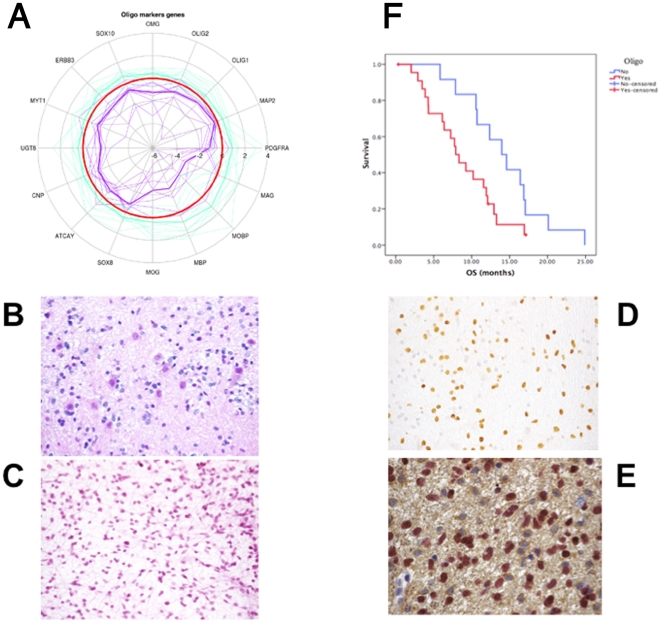
Description of the oligodendroglial/proneural type of DIPG. Panel A: Radial plot showing the expression of oligodendroglial markers in the two groups of DIPG, in log2 ratios related each other. The red circle represent the median expression level of the whole population of DIPG. Group 1 expresses higher levels of oligodendroglial markers than group 2 DIPG. Panel B: Morphological oligodendroglial differenciation in group 1 tumors (HES staining, ×40). Panel C: Morphological astrocytic differenciation in group 2 tumors (HES staining ×40). Panel D: Olig2 immunohistochemistry in a group 1 DIPG showing that probably not all cells in the biopsy are tumoral (×40). Panel E: Dual immunohistochemistry for Olig2 and GFAP showing that tumor cells in mitosis are GFAP negative but Olig2 positive (×100). Panel F: Overall survival of 55 DIPG according to the presence (red) or absence (blue) of oligodendroglial differenciation. Median OS was shorter in patients with oligodendroglial type of tumors (7.73 vs 12.37, p = 0.045, log rank test).

The group 1 of DIPGs as identified by gene expression profiling was characterized by the overexpression of oligodendroglial markers compared to group 2 (Figure 4A). Blinded morphological assessment revealed a significantly greater degree of oligodendroglial differentiation in these tumours compared with the mesenchymal group (Figure 4B and C). Strong expression of Olig2 by immunohistochemistry was seen in 13/13 tumors in this group *vs* 3/8 in group 2 tumours (p value = 0.003, chi square test with McNemar correction) (Figure 4D and E). Of note, *SOX10*, a known transcription factor involved in oligodendrogliogenesis [29], [30], was overexpressed in this subgroup compared to the other DIPG (log_2_ fold change 1.51 *vs* 0.21, adjusted p value = 0.0018). We used an extended cohort of 55 patients with histologically confirmed DIPG to study the impact of oligodendroglial differentiation on survival. Median overall survival of tumors with histological oligodendroglial features was 7.73 months *versus* 12.37 months for tumors that had predominantly astrocytic features (p = 0.045, log rank test) (Figure 4F).

**Figure 5:**
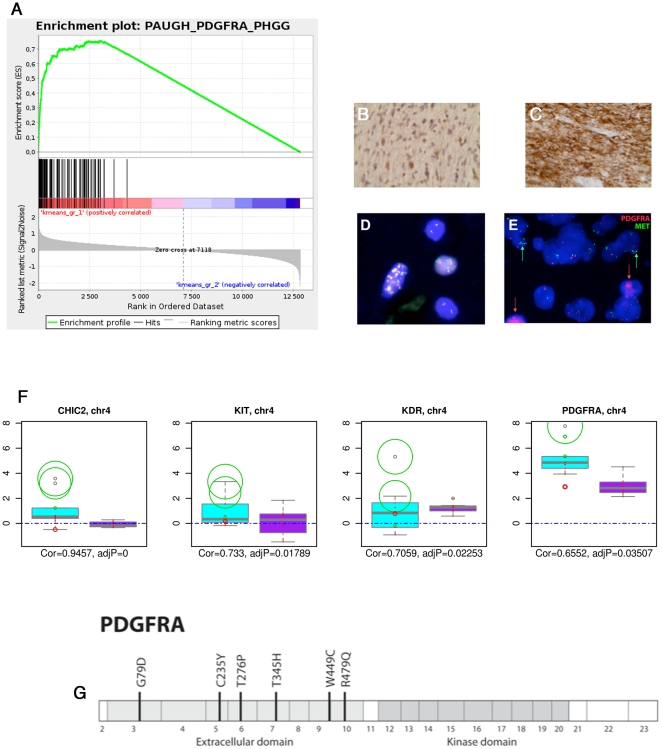
PDGFRA amplification/mutation is driving the oncogenesis of the oligodendroglial/proneural type of DIPG. Panel A: GSEA graph showing the enrichment of group 1 DIPG for the gene set describing the gene expression profile of PDGFRA amplified glioblastomas. Panel B: PDGFRA immunohistochemistry in the infiltrative part of a DIPG. Panel C: PDGFRA immunohistochemistry in the tumoral part of a DIPG. Panel D: FISH analysis of a DIPG using a FIP1L1/PDGFRA probe showing the amplification of the locus encompassing the two genes (most frequently seen). Panel E: Dual-FISH analysis of a DIPG with two probes one for PDGFRA and one for MET showing that the two oncogenes may be gained/amplified in different cells within the tumor. Panel F: Integrative genomic analysis using DR-Integrator (R package). Seven genes are present in the minimal common region (MCR) gained on chromosomal location 4q12 in DIPG. Boxplots represent the distribution of GE data and circles represent CNA data. The circles are centered on the corresponding GE measure on the distribution and their radii are proportional to the absolute value of CNA, red ones being losses and green ones gains. CNA and GE were highly correlated for four of these seven genes (*CHIC2, KIT, KDR, PDGFRA*). Panel G: Diagram of the PDGFRA gene showing the mutations discovered in DIPG samples and xenografts.

The gene expression profile of group 1 DIPG was significantly enriched for the gene set describing the signature of PDGFRA amplified gliomas described in the TCGA [24] and in children [8] (GSEA analysis: enrichment score 0.59, FDRq = 0.038, p nominal = 0.052) (Figure 5A). Although *PDGFRA* was overexpressed in most of the tumors compared to normal brain, this overexpression was significantly stronger in the group 1 tumours (p = 0.0055) (Figure 4A). This overexpression was confirmed by immunohistochemistry on an independent cohort in 9/15 cases that were screened for the target-driven exploratory study of imatinib in children with solid malignancies [20] (Figure 5B & C). Eight of nine cases with gain/amplification of *PDGFRA* detected by arrayCGH were found in this subgroup; these imbalances were confirmed by FISH in six samples for which the analysis was possible (Figure 5D). Simultaneous amplification of *PDGFRA* and *MET* was observed in 4 samples (Figure 5E). A similar observation of co-amplification of two RTK was observed in one patient for EGFR and PDGFRA (Figure S4). The minimal common region of the *PDGFRA* amplicon also contained *LNX1*, *RPL21P44*, *CHIC2*, *GSK2*, *KIT* and *KDR*. Integration of copy number with gene expression data demonstrated a high degree of correlation only for CHIC2, KIT, KDR and PDGFRA only (Figure 5F).

Sequencing the *PDGFRA* gene in an extended series of DIPG samples revealed no mutations in the kinase domains, known hotspots in other tumors such as gastro-intestinal stroma tumors [31]. By contrast, novel missense mutations were observed in the extracellular domains in 3/34 (8.8%) cases, and in a further two high grade gliomas established as primary xenografts (Figure 5G). One of the mutations in the IGRG82 pediatric glioma xenograft has been previously described in an adult glioblastoma (C235Y) (http://tcga-data.nci.nih.goc/docs/publications/gbm_exp/). Both mutant-positive cases for which gene expression data was available were part of the group 1 DIPG, and harboured *PDGFRA* gene amplification, as did the additional case in the extended series.

### DIPG subclasses signatures are enriched with genes of specific neural lineage

**Figure 6:**
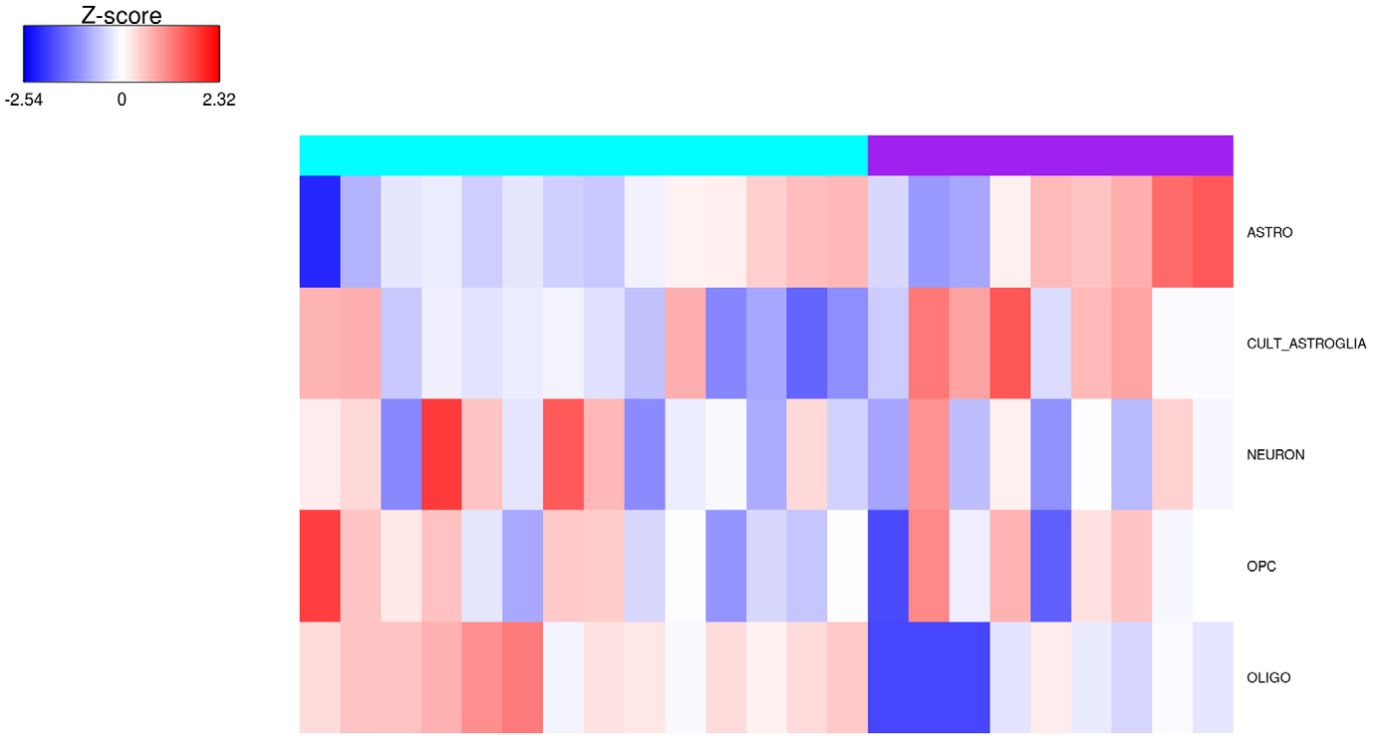
Comparison of gene expression signature of the two DIPG groups with specific neural lineages. A GSEA analysis was processed using the gene list previously described by Lei et al [32] and derived from the gene sets specifically enriched in astrocytes, oligodendrocytes, neurons, oligodendrocytes progenitors cells and cultured astroglial cells. Heatmap of the enrichment scores of each DIPG sample is represented with a red to blue color scale shows the range from the highest to lowest enrichment score.

We conducted a GSEA to compare the GE profile of the two groups of DIPG to the gene list generated from 5 neural linages isolated from mouse brain developped by Lei et al [32] from the transcriptome database of Cahoy et al [33]. Tumors of group 1 DIPG were enriched with the gene signature of mature oligodendrocytes and to a lower extent with the one of oligodendrocyte precursor cells (OPC), ressembling in this respect to the proneural class of GBM glioblastoma (Figure 6). Conversely tumors of group 2 DIPG were enriched with the gene signatures of astrocytes and cultured astroglia (Figure 6). This later group of DIPG shared in this respect the GE signatures of the mesenchymal and classical classes of GBM that were enriched with the gene list of cultured astroglia and astrocytes, respectively.

## Discussion

In this study, we report the first comprehensive genomic analysis of DIPG samples taken at diagnosis, and identify key biological features which distinguish them from other pediatric supratentorial HGG. The gene expression signatures associated with the location of a tumour was associated with differential reprogramming of embryonic signaling organizers, reflecting the discrete developmental origins of HGG presenting in different locations in the brain. Furthermore, our data indicate that DIPG arise from two distinct oncogenic pathways. The first group of DIPG exhibits an oligodendroglial phenotype associated with PDGFRA gain/amplification. Its gene expression profile is enriched for the proneural and PDGFRA-amplified glioma signatures. It comprises the most clinically aggressive tumours, independent of histological grade. The second group of DIPG exhibits a mesenchymal and pro-angiogenic phenotype orchestrated by a similar transcriptional module to that recently described in adult glioblastomas. These data greatly prolong our understanding of the molecular pathogenesis of pediatric DIPG and HGG, and have significant implications the future clinical management of children with these tumours.

### DIPG Represent a Biologically Distinct Group of HGG in Children

Pediatric DIPG and supratentorial high-grade gliomas, although harboring overlapping patterns of chromosomal imbalances, could be clearly differentiated through their gene expression signatures. Among the most differentially expressed genes with respect to tumour location, we identified numerous homeobox and HLH genes that were associated with brainstem tumours, and likely represent embryonic signaling organizers that have undergone transcriptional reprogramming during oncogenesis. The concept of location driving tumorigenesis in the brain [34] has been applied to other tumor types like ependymoma [35]–[38] and pilocytic astrocytomas [39], where developmentally-restricted gene expression signatures could be related to the site of tumor growth. Interestingly, genes found to be overexpressed in DIPG compared to supratentorial HGG, such as *LHX2* and *IRX2*, have been previously described to be overexpressed in posterior fossa pilocytic astrocytomas and ependymomas compared to their supratentorial counterparts [36], [38], [39]. The converse may also be true, with *FOXG1* and *ZFHX4* found to be upregulated in supratentorial HGG compared with DIPG, similar to data from ependymomas and pilocytic astrocytomas [36], [38], [39]. This suggests that there may be a common gene expression pattern related to the location and developmental origin of glial tumors irrespective of the histological diagnosis. Moreover, among the genes whose expression distinguished DIPG from the HGG in other location, we identified several genes involved in the SHh pathway such as PTCH1, GLIS1, GJA1, SLC1A6, KCND2, PENK, GAD1 (see Table S2) already shown to be upregulated in mouse models [40]. This is in line with data from Monje et al. who have recently shown the possible role of the Sonic Hedgehog pathway in the oncogenesis of DIPG [41].

Of particular significance was the similarity of gene expression profiles of HGG arising in the midline/thalamus with DIPG, and their distinction from hemispheric tumours, likely indicating expansion from closely-related precursor populations, in these tumours for which the cell(s) of origin are yet not known. Although the adoption of different treatment strategies for DIPG and supratentorial HGG is well-established in clinical practice, the biological resemblance of midline/thalamic tumors and DIPG raises questions regarding the management of these specific neoplasms, currently focused on strategies designed for supratentorial HGG [42].

### Mesenchymal transition with a stem cell-like phenotype is the hallmark of a subset of DIPG

While a mesenchymal phenotype appears only infrequently represented in pediatric supratentorial HGG [8], almost half of the pediatric DIPG were characterized by the overexpression of biomarkers of mesenchymal transition, stemness and a hypoxia-induced angiogenic switch. The transcriptional module driving the mesenchymal gene expression signature in adult glioblastoma [25] was also specifically overexpressed in this group compared to the proneural group. The acquisition of a mesenchymal phenotype [43], stemness [44], as well as the expression of hypoxia-related genes [45], [46] have been associated with resistance to treatment including radiotherapy. The enhanced self-renewing capability of this subtype of DIPG further points to a distinct development lineage from the more differentiated PDGFRA-driven DIPG. In this respect, the higher expression of *STAT3* in the mesenchymal type of DIPG compared to the proneural one may play a key role in their opposite differentiation. Indeed, *STAT3* elimination promote neurogenesis and inhibit astrogenesis in neural stem cell, ie the phenotype of group 1 DIPG [47]. Glioma stem cells are associated with a perivascular niche, and appear to modulate vascular proliferation via VEGF, itself regulated via the HIF pathway. These three phenomenons are closely interrelated in several cancers including glioblastoma [48]–[51], and open the possibility that agents which target angiogenesis and/or drive differentiation of tumour stem cells may find application in this subset of DIPG to increase the antitumor effects of ionizing radiation.

Despite the involvement of Ras pathway in epithelio-mesenchymal transition via SNAI2 [52] and its link with worse outcome of pediatric HGG [53], we did not find a correlation between *H-RAS* gain/amplification and its gene expression, nor activating mutations in the *RAS* genes including *BRAF* V600E already described in some pediatric supratentorial gliomas [27], again highlighting differential oncogenic mechanisms in DIPG compared to other pediatric HGG.

### Proneural and oligodendroglial differentiation associated with PDGFRA amplification

We have identified through unsupervised gene expression clustering a group of DIPG characterized by a ‘proneural’ phenotype, an oligodendroglial differentiation, and *PDGFRA* amplifications/mutations. Moreover, the gene expression profile of group 1 DIPG was significantly enriched with genes describing the signature of PDGFRA amplified gliomas [8], [24] supporting the hypothesis that PDGFRA amplification is associated with a robust gene expression profile across tumor location and patient's age. This association has been previously described in adult tumors [43], [54]–[56], and include the expression of genes involved in neurogenesis and oligodendrocyte development, such as *Olig* transcription factors, *Nkx2.2*, *PDGFRA* and *SOX10* [57]. DIPG with oligodendroglial phenotype and Olig2 overexpression exhibited an even worse evolution and resistance to radiation than the other DIPG in our series. This could be explained by the recent findings that the central nervous system-restricted transcription factor Olig2 opposes p53 response to genotoxic damage in neural progenitors and malignant glioma [58]. This is however in contrast with the adult gliomas where oligodendroglial differentiation and proneural phenotype are linked with a better prognosis [24]. Moreover, we did not observe IDH1/2 mutation in 10 DIPG [59] while in adult proneural gliomas IDH1 mutations are frequent [24]. In pediatric gliomas, IDH1/2 mutations are almost exclusively seen in adolescents [59], [60] who indeed do not represent the target population of DIPG. The presence of IDH1 mutation in tumors from adolescents was not correlated with an oligodendroglial phenotype in our cohort of pHGG previously published [59]. Together with the fact that the group 1 DIPG is enriched preferentially with the signature of mature oligodendrocytes rather than oligodendrocyte progenitor cells, these data could suggest that this group of DIPG could be developed from a different oligodendroglial cell than their adult counterpart. This would be in line with the rarity of 1p19q co-deletion in pediatric gliomas with oligodendroglial features.

Integrative genomics showed that the gene expression of this group of DIPG was driven by copy number changes on the contrary to the other DIPG suggesting that chromosomal instability plays an important role in the phenotype of these tumors. Conversely, gene expression in the other group of DIPG may be more driven by epigenetic changes.

We found 28% (9/32) of PDGFRA gains or amplifications, all but one being included in the group 1 defined by unsupervised gene expression clustering. The PDGF autocrine/paracrine loop has been frequently implicated in oligodendrogliomas [61] and has been used to create preclinical models of glioma [62], [63], including brainstem tumors [64], [65]. PDGFRA amplification has been shown to be more frequent in pediatric HGG than in adult ones [8] and a recent report found PDGFRA gain or amplification in four out of eleven post-mortem samples of DIPG [10]. In one of our previous study, PDGFRA protein was also more frequently detected by IHC in DIPG than in other pediatric HGG [20].

We identified 10% of pediatric DIPG to harbor *PDGFRA* missense mutations, considerably more frequently than the 2/206 (1%) reported in adult GBM (http://tcga-data.nci.nih.goc/docs/publications/gbm_exp/). These mutations were located in exons coding for the extracellular domains of the protein, potentially disrupting ligand interaction, but not in the tyrosine-kinase domain. Their oncogenic role can be suspected, especially as they are found exclusively in concert with gene amplification. Similarly, mutations have been found in the ectodomain but not in the tyrosine-kinase domain of *EGFR* gene in adult GBM [66]; these mutations were shown to be oncogenic. Moreover, similar to EGFRvIII mutants, deletions in the extracellular domain of PDGFRA have been already reported in as many as 40% of glioblastomas with PDGFRA amplification and were associated with increased tyrosine-kinase activity [67]. Unfortunately, the assay used for PDGFRA sequencing did not allow us to exclude the possibility of in frame deletions and this would need further analysis on new samples.

### Translational implications of targeting genomic alterations in DIPG

Lack of insight into disease mechanisms impeded the development of effective therapies in DIPG for years, with the selection of therapeutic agents to be used in conjunction with irradiation determined empirically or based on their efficacy in adult high-grade gliomas. Changing the paradigm of the treatment of this disease requires a better understanding of the key biological events driving this type of neoplasm. Our clinical and biological program allowed us to discover new potential therapeutic targets previously overlooked or ignored. For the first time, rationale design of trials with targeted therapies could be implemented in the armentarium against these aggressive neoplasms. PDGFRA indeed seems to be the most exciting target given also the existence of several inhibitors with a known toxicity profile in children, including patients with DIPG at relapse [20] or at diagnosis after irradiation [68]. Despite significant drug concentrations reached inside the glioblastoma [69], imatinib has shown limited efficacy in recurrent or newly diagnosed glioblastoma in adults [70] and response to the drug was not increased in patients with PDGFRA immunopositivity [71]. No information on the histology of the brainstem tumors was available in the Pediatric Brain Tumor Consortium (PBTC) phase II trial of imatinib [68] where most of the patients with brainstem gliomas received indeed the drug after the completion of their radiotherapy schedule. In a recent study of the ‘Innovative Therapies in Children with Cancer’ consortium, where imatinib was only given to patients with proven PDGFRA, PDGFRB or KIT over expression determined by immunochemistry [20], one child with recurrent DIPG harboring PDGFRA expression in 50% of the cells in the biopsy showed a sustained objective response (minus 31% for tumor size) for a period of ten months. Identifying the key predictive markers for efficacy of targeted agents will be a vital step in translating genomic data to the clinic, particularly where specific activating mutations are identified. The literature [70], [71] indicates however that the effect of imatinib as single agent is limited and that combination with other agents such as irradiation should be considered [72], [73]. In addition, insufficient drug penetration in the brain and in some part of the tumor may explain these disappointing results. Enhanced delivery would then need either blood to brain barrier opening [74] or P-gp and ABCG2 inhibition [75]. In this respect the DIPG orthotopic models newly described [63], [64] will be valuable tools to study the appropriate way to deliver these drugs in addition to help our understanding of the disease. Combinatorial targeted approaches may also be valid given the observation of multiple oncogenic alterations activating the same downstream signaling cascades [76]. Our finding of simultaneous amplification of PDGFRA and MET in a subset of DIPG, for example, may justify the use of multikinase inhibitors or combinations of TKI, as has been demonstrated for pediatric glioblastoma cells *in vitro* [77].

Our integrated genetic profiling of diagnostic DIPG has identified two biologically and clinically distinct groups of DIPG, with clear differences from hemispheric HGG, and with likely differential treatment strategies warranted. These data highlight the importance of biologically driven guidance for novel therapeutic intervention in these currently untreatable tumors, and argue for the systematic biopsy of these lesions in order to facilitate this, in addition suggesting that some supratentorial deep-seated infiltrating HGG of the deep grey nuclei may deserve a similar approach.

## Materials and Methods

### Tumor and Nucleic Acids extraction

Tumor samples and clinical information were collected with written informed consent (see Supporting Information S1) of the parents/guardians before inclusion into protocols approved by the Internal Review Board of the Necker Sick Children's Hospital in Paris and the Gustave Roussy Cancer Institute in Villejuif [corresponding to two phase I/II trials, see references 20 and 21]. Only patient with classical diagnostic features of DIPG were included: 1) short clinical history of less than three months, 2) infiltrating neoplasm centered on the pons and involving at least 50% of the anatomical structure, 3) histology excluding a pilocytic astrocytoma or ganglioglioma.

Tumor biopsies were snap frozen in liquid nitrogen in the operating room to ensure preservation of high quality RNA, ground to powder and then RNA and DNA were extracted following two different protocols according to their respective efficiency: Rneasy Micro Kit (Qiagen) and/or TRIzol reagent (Invitrogen).

### Microarray Analyses

DNA and RNA microarray hybridizations were carried out by the Functional Genomics Platform of the Integrated Research Cancer Institute in Villejuif (http://www.igr.fr/en/page/integrated-biology_1529) using the Agilent 44 K Whole Human Genome Array G4410B and G4112F, respectively (http://www.agilent.com). The microarray data related to this paper are MIAME compliant and the raw data have been submitted to the Array Express data repository at the European Bioinformatics Institute (http://www.ebi.ac.uk/arrayexpress/) under the accession number E-TABM-1107.

### Bioinformactic Analyses

Raw copy number ratio data were transferred to the CGH Analytics v3.4.40 software for further analysis with the ADM-2 algorithm (http://www.agilent.com). A low-level copy number gain was defined as a log 2(ratio) >0.3 and a copy number loss was defined as a log 2(ratio) <−0.3. A high-level gain or amplification was defined as a log 2(ratio) >1.5. Minimum common regions (MCR) were defined as chromosome regions that show maximal overlapping aberrations across multiple samples with the STAC v1.2 software [78]. Probe-level measurement MCRs do not include all genes that are altered within a given aberrant region in a particular tumor but define the recurrent abnormalities that span the region.

Raw gene expression data using normal brainstem as reference were transferred into R software for statistical analysis. In order to discover groups in GE data set, the k-means algorithm from R software has been run for two to five groups on the entire dataset. Then for each clustering the BIC value was calculated, according to Guillemot et al [22], in order to determine the best one, which was the one with two groups. GSEA analysis [23] was performed with the pre-ranked tool on gene list ranked by increasing FDR adjusted p-values, for each contrast of interest, with default parameter values. A nominal False Discovery Rate (FDR) of <0.25 was considered statistically significant for GSEA. We ran GSEA analysis with t-test option as metric parameter.

For integrative genomics analysis, we used the DR-Integrator package for R [79].

### Fluorescent In Situ Hybridization

FISH was performed from formalin-fixed-paraffin-embedded (FFPE) tumor samples or frozen tumor touch slides for the xenografts. The FIP1L1/PDGFRA (Q-biogen/MP Medicals) and LSI EGFR (Vysis/Abbot) were used according to the manufacturer's instructions. PDGFRA and MET probes were labelled from BAC-clones RP11-58C6 and RP11-819D11 (PDGFRA) and RP11-165C4 and RP11-951I21 (MET) using the Bioprime kit (Invitrogen) and DIG-6-dUTP (Roche). Slides were pre-treated in 0.2 M HCl, 8% sodium thiocyanate and 0.025% pepsin. Probes were hybridised overnight at 37C. Slides were washed and incubated with conjugates streptavidin-Cy3 (Invitrogen) and anti-DIG-FITC (Roche).

### Mutation screening of selected genes

For direct sequencing, the exon 15 of *BRAF* and all the individual exons of *PDGFRA* were PCR amplified using Taq DNA polymerase (Invitrogen) and primers that can be provided upon request. PCR products were sequenced with BigDye v3.1 and run on an AB3730 genetic analyser (Applied Biosystems). Traces were analysed using Mutation Surveyor software (Softgenetics). The effect of the mutations on the protein structure was predicted using Polyphen (http://genetics.bwh.harvard.edu/pph/) and SIFT (http://sift.jcvi.org/) databases.

### Histology and Immunohistochemistry on Primary Tumor Material

Tumor histology was reviewed by PV. Tumors were classified and graded according to the 2007 WHO classification. Representative formalin-zinc (formol 5%; Zinc 3 g/L; sodium chloride 8 g/L) fixed sections were deparaffinized and subjected to a Ventana autostainer (BenchMark XT, Ventana Medical system, Tucson, USA) with a standard pretraitement protocol included CC1 buffer for MIB (KI-67) and P53. A semi-automatised system using a microwave antigen retrieval (MicroMED T/T Mega; Hacker Instruments & Industries, Inc., Winnsboro, SC) for 30 minutes at 98°C (manufacturer recommendations) and the RTU Vectastain Universal detection system (Vector laboratories, Burligame, CA, USA) for Olig2. Sections were then incubated with various commercial monoclonal primary antibodies against Olig2 (AF 2418, 1/150, R/D system, CA, USA), P53 (DO-1, 1/1, Ventana) and MIB-1 (1/100; Dako, Glostrup, Denmark). Diaminobenzidine was used as the chromogen. A minimal threshold at 10% of the total stained tumor cells served as a cut-off for defining the p53-positive status. A MIB-1 labeling index (MIB-1 LI) was obtained by counting the number of MIB-1-positive tumor cells in regions with the maximum number of labeled tumor cells. Ten microscopic high-power field sets were counted, and the MIB-1 LI was computed as a percentage of immunopositive cells from the total cells counted in selected fields. Light microscopic images were digitally captured using a Nikon eclipse E600 microscope (Nikon, Tokyo, Japan) equipped with Nikon DXM 1200 Digital camera. Photomicrographs were assembled for illustrations using the Adobe Photoshop version 7.0.1 software (Adobe, San Jose, California, USA).

## Supporting Information

Figure S1 **DIPG are different from supratentorial high-grade gliomas in children. Panel A: example of a biopsy sampling in a patient with DIPG.** A maximum of 8 core biopsy samples can be obtained per patient. **Panel B: heatmap of the unsupervised hierarchical clustering of 29 DIPG.** From the 32 available samples, two had a completely flat profile and one was of unsufficient quality. The analaysis was then run on 29 samples. Gains are represented in green (the intensity being correlated to the log2ratio) and amplifications as blue dots. Losses are represented in red (the intensity being correlated to the log2ratio). The lower panel indicate the general profile of genomic imbalances encountered in the 32 samples, y axis scale being the frequency of the aberrations. The colored right panel shows the profile of each individual sample and the black & white right panel shows the percentage of the genome with imbalances. **C: overall survival of the patients with CGHarray data according to the loss or the persistence of the** ***TP53*** **locus.** Overall survival was significantly lower in patients with *TP53* gene loss (p = 0.01, log-rank test). **D: principal component analysis (PCA) of pediatric high-grade gliomas (HGG) CGHarray data irrespective of their location.** Hemispheric HGG are indicated in yellow, midline HGG are indicated in grey and brainstem HGG or DIPG are indicated in pink. All the probes passing the quality control were used for the analysis. **E: box-plots comparing the expression of some of the key regulators of brainstem embryogenesis in DIPG (pink) and supratentorial HGG (yellow).** The adjusted p-value of the comparison is given in the upper left corner of each panel. All values are given relative to the expression found in normal adult brainstem.
(TIFF)

Figure S2 **DIPG comprises two biological subgroups with distinct survival and pathological characteristics. A: Identification of the most optimal Bayesian Information Criterion (BIC) value.** The most optimal BIC value was obtained using the class prediction algorithm of Guillemot et al. (BIOTECHNO'08). The graphs show that the accuracy of class prediction did not improve with increasing number of groups. **B: Integrative analysis of genomic and gene expression data.** When considering all DIPG samples from whom both GE and CGHarray data were available, the expression of 1460 genes (ie 6% of the genome) was significantly correlated with copy numbers. The cheese-plots of the 20 genes with the highest correlation are provided. Complete data set is available upon request.
(TIFF)

Figure S3 **Mesenchymal transition and a pro-angiogenic switch define a subset of DIPG. A: The master epithelial to mesenchymal transition regulators,** ***SNAI1*** **and** ***SNAI2/Slug*** **are upregulated in a subset of DIPG.** The box-plots of the two DIPG subgroups identified are shown in purple and brown respectively. Gene expression are given compared to normal adult brainstem. The p-value is indicated for each gene in the upper right corner of the panel. **B: Angiogenic markers are overexpressed in a subgroup of DIPG.** The two different subgroups of DIPG are represented in purple and light green. The p-value is indicated for each gene in the upper right corner of the panel. Gene expression are given compared to normal adult brainstem. **C: Stem cell markers are overexpressed in a subgroup of DIPG.** The two different subgroups of DIPG are represented in purple and cyan. The p-value is indicated for each gene in the upper left corner of the panel. Gene expression are given compared to normal adult brainstem. **D: Gene expression profiling of one of the DIPG stem cell cultures.** Principal component analysis of one of the DIPG stem cell cultures together with all the primary DIPG samples.
(TIFF)

Figure S4 **Amplification of multiple RTK in the same tumor.** Example of a DIPG sample for which simultaneous amplification of PDGFRA and EGFR could be observed by FISH.
(TIFF)

Table S1 (XLSX)

Table S2 (XLS)

Table S3 (XLS)

Supporting Information S1 (PDF)
