# Supplementary figures and images for: Mesenchymal Transition and PDGFRA Amplification/Mutation Are Key Distinct Oncogenic Events in Pediatric Diffuse Intrinsic Pontine Gliomas
Source: PLoS One. 2012 Feb 28;7(2):e30313. doi: 10.1371/journal.pone.0030313 (PMC3289615; doi:10.1371/journal.pone.0030313)

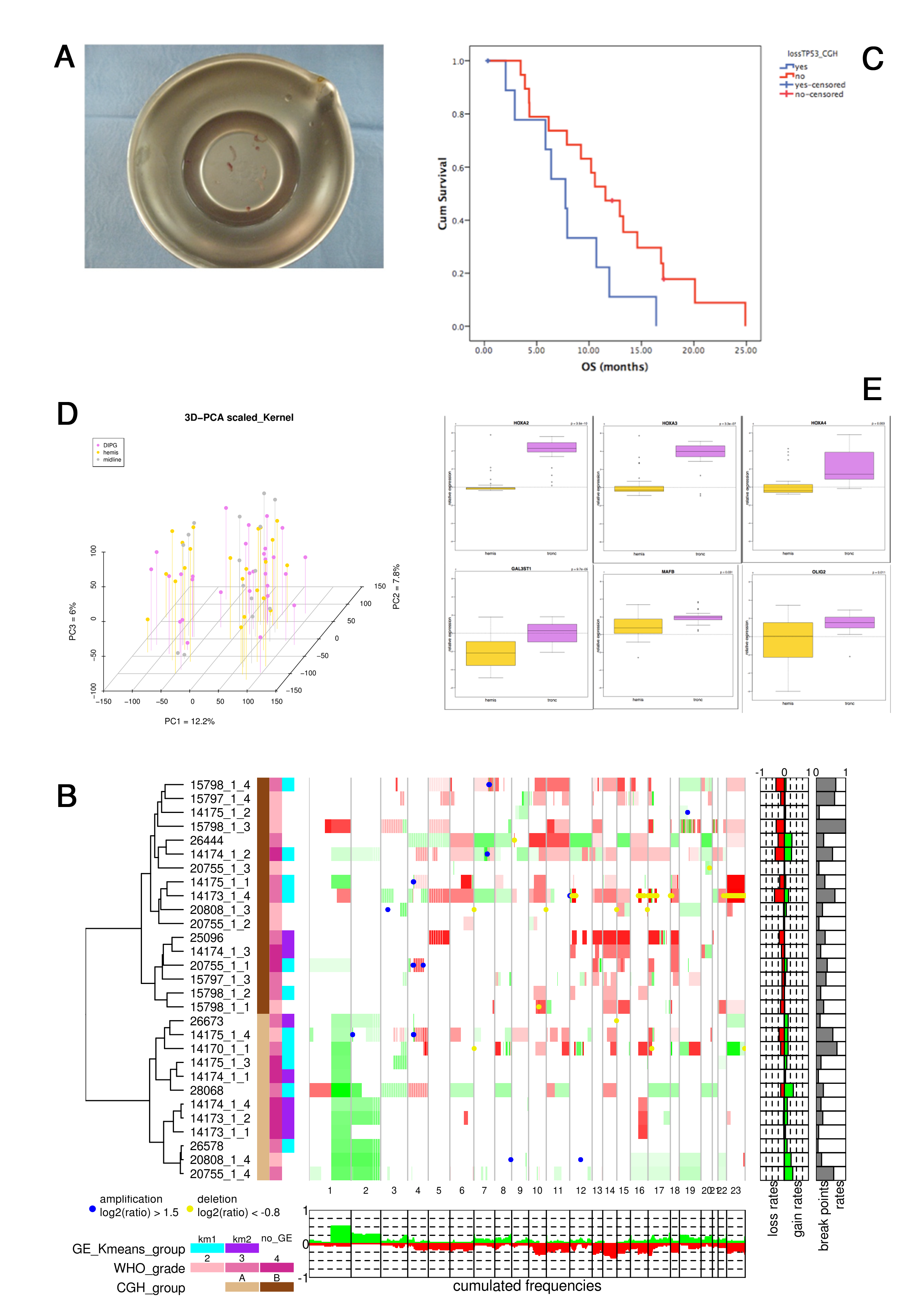

Supplement: Figure S1 — DIPG are different from supratentorial high-grade gliomas in children. Panel A: example of a biopsy sampling in a patient with DIPG. A maximum of 8 core biopsy samples can be obtained per patient. Panel B: heatmap of the unsupervised hierarchical clustering of 29 DIPG. From the 32 available samples, two had a completely flat profile and one was of unsufficient quality. The analaysis was then run on 29 samples. Gains are represented in green (the intensity being correlated to the log2ratio) and amplifications as blue dots. Losses are represented in red (the intensity being correlated to the log2ratio). The lower panel indicate the general profile of genomic imbalances encountered in the 32 samples, y axis scale being the frequency of the aberrations. The colored right panel shows the profile of each individual sample and the black & white right panel shows the percentage of the genome with imbalances. C: overall survival of the patients with CGHarray data according to the loss or the persistence of the TP53 locus. Overall survival was significantly lower in patients with TP53 gene loss (p = 0.01, log-rank test). D: principal component analysis (PCA) of pediatric high-grade gliomas (HGG) CGHarray data irrespective of their location. Hemispheric HGG are indicated in yellow, midline HGG are indicated in grey and brainstem HGG or DIPG are indicated in pink. All the probes passing the quality control were used for the analysis. E: box-plots comparing the expression of some of the key regulators of brainstem embryogenesis in DIPG (pink) and supratentorial HGG (yellow). The adjusted p-value of the comparison is given in the upper left corner of each panel. All values are given relative to the expression found in normal adult brainstem. (TIFF) [file pone.0030313.s001.tiff]

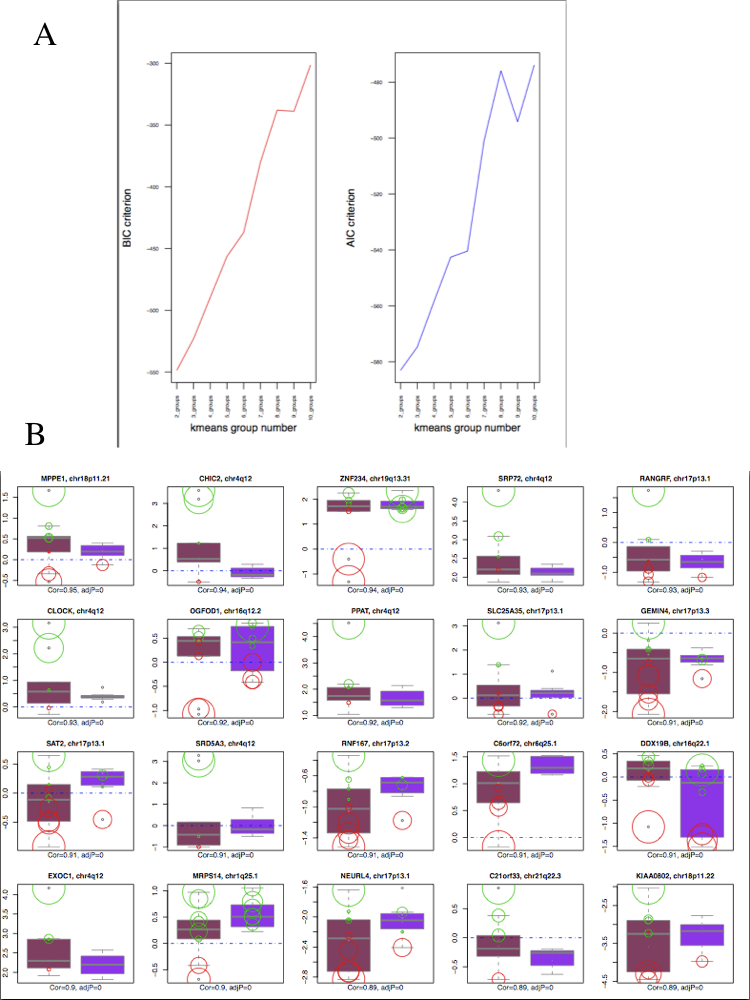

Supplement: Figure S2 — DIPG comprises two biological subgroups with distinct survival and pathological characteristics. A: Identification of the most optimal Bayesian Information Criterion (BIC) value. The most optimal BIC value was obtained using the class prediction algorithm of Guillemot et al. (BIOTECHNO'08). The graphs show that the accuracy of class prediction did not improve with increasing number of groups. B: Integrative analysis of genomic and gene expression data. When considering all DIPG samples from whom both GE and CGHarray data were available, the expression of 1460 genes (ie 6% of the genome) was significantly correlated with copy numbers. The cheese-plots of the 20 genes with the highest correlation are provided. Complete data set is available upon request. (TIFF) [file pone.0030313.s002.tiff]

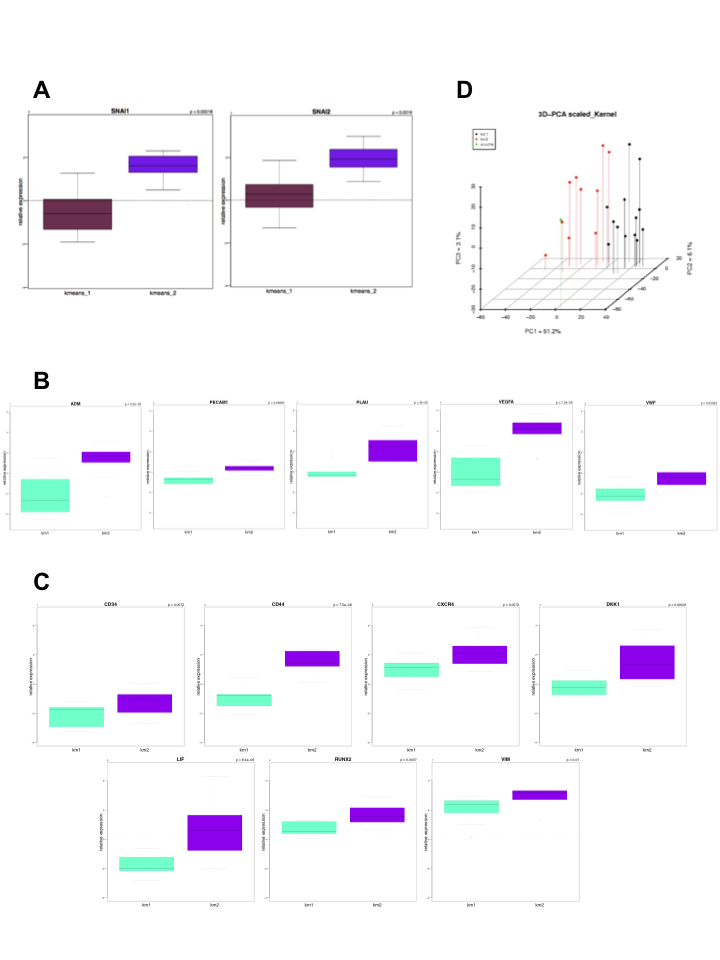

Supplement: Figure S3 — Mesenchymal transition and a pro-angiogenic switch define a subset of DIPG. A: The master epithelial to mesenchymal transition regulators, SNAI1 and SNAI2/Slug are upregulated in a subset of DIPG. The box-plots of the two DIPG subgroups identified are shown in purple and brown respectively. Gene expression are given compared to normal adult brainstem. The p-value is indicated for each gene in the upper right corner of the panel. B: Angiogenic markers are overexpressed in a subgroup of DIPG. The two different subgroups of DIPG are represented in purple and light green. The p-value is indicated for each gene in the upper right corner of the panel. Gene expression are given compared to normal adult brainstem. C: Stem cell markers are overexpressed in a subgroup of DIPG. The two different subgroups of DIPG are represented in purple and cyan. The p-value is indicated for each gene in the upper left corner of the panel. Gene expression are given compared to normal adult brainstem. D: Gene expression profiling of one of the DIPG stem cell cultures. Principal component analysis of one of the DIPG stem cell cultures together with all the primary DIPG samples. (TIFF) [file pone.0030313.s003.tiff]

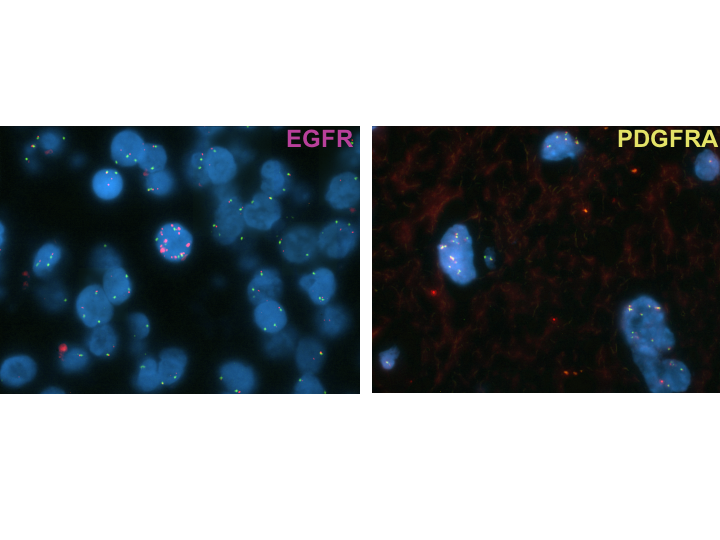

Supplement: Figure S4 — Amplification of multiple RTK in the same tumor. Example of a DIPG sample for which simultaneous amplification of PDGFRA and EGFR could be observed by FISH. (TIFF) [file pone.0030313.s004.tiff]
