# Supplementary material for: Mesenchymal Transition and PDGFRA Amplification/Mutation Are Key Distinct Oncogenic Events in Pediatric Diffuse Intrinsic Pontine Gliomas
Source: PLoS One. 2012 Feb 28;7(2):e30313. doi: 10.1371/journal.pone.0030313 (PMC3289615; doi:10.1371/journal.pone.0030313)
Supplement: Supporting Information S1 — (PDF) [file pone.0030313.s008.pdf]

ASSISTANCE PUBLIQUE HÔPITAUX DE PARIS

Formulaire d'information et consentement d'un patient mineur sur l'utilisation à des fins de recherche scientifique du sérum, plasma, cellules ou tissus, incluant les tissus tumoraux prélevés à l'occasion des soins

Madame, Monsieur,

Votre enfant est suivi par le Docteur..,
consultant(e) à l'Assistance Publique – Hôpitaux de Paris.

Dans le cadre de la prise en charge de votre enfant, les médecins peuvent être amenés pour assurer ses soins, à prélever du sang, des cellules ou des tissus qui seront stockés au Centre de Ressources Biologiques de l'hôpital Necker Enfants Malades. Ces prélèvements sont effectués pour établir le diagnostic de sa maladie, la réalisation et le suivi de son traitement, ce dernier incluant dans certains cas une greffe de cellules. Ils sont utilisés dans son intérêt, pour permettre les meilleurs soins possibles.

Pour assurer correctement les soins de votre enfant, ses prélèvements seront conservés par congélation. C'est une technique très performante qui permet de les garder de nombreuses années dans de très bonnes conditions de sécurité. La structure chargée de cette mission est le Centre de Ressources Biologiques de l'hôpital Necker Enfants Malades - Assistance Publique – Hôpitaux de Paris : C'est la bibliothèque des prélèvements biologiques.

Une partie des prélèvements de votre enfant peut n'être pas nécessaire pour assurer ses soins et son traitement. Celle-ci présente un grand intérêt pour la réalisation d'études scientifiques. Nous souhaiterions pouvoir, avec votre accord, utiliser cette partie dans une finalité de recherche. Bien entendu, l'utilisation de ses prélèvements dans cette finalité ultérieure de recherche n'a aucune conséquence sur les soins qui lui sont prodigués.

Lorsque l'utilisation des prélèvements est susceptible de comprendre un examen des caractéristiques génétiques de votre enfant, vous serez informé de la nature et de la finalité de l'examen et votre consentement et celui de votre enfant, s'il est apte à exprimer sa volonté, sera recueilli par écrit préalablement à la réalisation de l'examen.

Les programmes de recherche développés à l'hôpital Necker Enfants Malades - Assistance Publique – Hôpitaux de Paris visent à élucider les mécanismes des maladies, ou la mise en place de traitements nouveaux. Ces programmes peuvent être mis en place dans le cadre du Programme Hospitalier de Recherche Clinique financé par le Ministère de la Santé, des appels d'offres à des programmes de recherche financés par l'Assistance Publique – Hôpitaux de Paris, l'INSERM, le CNRS ou encore dans des programmes de collaboration de recherche avec l'Industrie Pharmaceutique.

Les résultats de ces recherches peuvent être l'objet de publications dans les revues scientifiques.
Les connaissances et innovations qui sont mises en évidence peuvent aussi donner lieu à des dépôts de brevets. Ces brevets peuvent ensuite être cédés à des industries pharmaceutiques intéressées dans le développement de médicaments ou de tests innovants. Lorsque des bénéfices financiers sont dégagés grâce à la valorisation de ces résultats, c'est à dire un retour financier vers l'Assistance Publique - Hôpitaux de Paris, organisme public, ils seront comme le prévoit le code de la propriété intellectuelle, en partie réinvestis par l'Assistance Publique – Hôpitaux de Paris dans le financement de ses appels d'offres à des programmes de recherche.
Comme le prévoit la Directive européenne 98/44/CE sur les inventions de biotechnologie, un brevet ne peut être déposé suite à une découverte réalisée sur un matériel biologique qu'avec l'accord du donneur.

Pour toutes ces raisons, nous souhaitons recueillir votre accord et celui de votre enfant, s'il est apte à exprimer sa volonté, à la réalisation de recherches à partir de ses prélèvements. Vous pouvez exprimer votre consentement en remplissant le volet 3 de ce document d'information.

Après l'avoir daté et signé, nous vous remercions de le remettre à la fin du passage de votre enfant à l'hôpital.

Bien entendu, vous ou votre enfant, avez toujours la possibilité de revenir sur votre accord, sans avoir à expliquer les raisons ; il vous suffira de nous le faire savoir.

Vous ou votre enfant, pouvez exprimer votre refus de la même manière.
Si vous ne nous remettez pas ce document, votre absence de réponse sera considérée comme un refus, et les prélèvements de votre enfant ne pourront pas être utilisés dans le cadre de recherches.

Pour toute recherche, il est également nécessaire de disposer des données cliniques concernant votre enfant. Comme vous le savez, celles-ci sont conservées dans son dossier médical et gérées informatiquement ; le traitement automatisé des informations concernant votre enfant a été autorisé par la Commission Nationale de l'informatique et des Libertés, ainsi que l'indique le livret d'accueil qui vous a été remis lors de son séjour.

De même qu'une partie de prélèvement de votre enfant est utile pour les recherches scientifiques, les données médicales le concernant fournissent les informations médicales nécessaires pour mener à bien les travaux effectués sur les prélèvements. Les nouvelles connaissances sur les maladies sont le plus souvent produites grâce au rapprochement des résultats d'analyses biologiques avec les observations cliniques des maladies et de leur évolution.

Vous ou votre enfant, pouvez vous opposer à l'utilisation des données médicales personnelles du dossier de votre enfant à de fins de recherche, en exprimant votre refus.
Si vous ou votre enfant, exprimez votre refus, ses données médicales ne seront utilisées dans aucune recherche nécessitant leur analyse.
Bien entendu, vous n'avez pas à expliquer les raisons de ce choix.

Dans l'hypothèse où vous ou votre enfant, n'avez pas exprimé de refus, et si les données médicales de votre enfant sont utilisées dans le cadre d'un projet scientifique, elles sont rendues anonymes avant leur utilisation.

Lorsque vous aurez remis ce document, vos choix sur votre accord ou votre opposition à l'utilisation du surplus du matériel biologique et des informations médicales à des fins de recherche, de votre enfant, seront insérés dans son dossier médical.

Sera donc indiqué, selon les cas, votre accord ou votre refus ou celui de votre enfant, pour l'utilisation ses prélèvements ainsi qu'un éventuel refus pour l'utilisation de ses données médicales.

Madame, Monsieur,
Quelle que soit votre décision, nous tenons à vous remercier de l'attention que vous avez portée à la lecture de ce document.

Si les explications qui vous ont été fournies vous semblent insuffisantes, ou si vous souhaitez obtenir des informations supplémentaires vous pouvez vous adresser au médecin qui soigne votre enfant, ou par courrier en nous écrivant à :

Centre de Ressources Biologiques
Hôpital Necker Enfants Malades
Assistance Publique - Hôpitaux de Paris
149 rue de Sèvres 75743 Paris Cedex 15

<u>Note importante :</u>
Ce document écrit vient en complément d'une information orale qui doit vous être fournie par un médecin au cours d'un entretien particulier.

- ***Joindre l'original de ce document au prélèvement,***
- ***Conserver le feuillet n°2 dans le dossier médical,***
- ***Remettre le feuillet n°3 au patient.***

Etiquette du patient à coller ***sur les 3 feuillets ou***	Partie réservée au Centre de Ressources Biologiques
Nom patronymique	
Prénom	Numéro d'inscription : ....
Date de naissance	

CONSENTEMENT

Nous avons pris connaissance du document nous informant que le Centre de Ressources Biologiques de l'hôpital Necker Enfants Malades - Assistance Publique - Hôpitaux de Paris a pour mission de conserver le sérum, le plasma, les cellules, les tissus, incluant des tissus tumoraux prélevés dans l'intérêt de mon enfant pour lui permettre de recevoir des soins adaptés à sa maladie, mais que la partie de ces prélèvements non utilisée pour le soigner peut également être utile à la réalisation de recherches de nature scientifique.

Nous savons qu'ils pourront être utilisés par des chercheurs travaillant à l'Assistance Publique – Hôpitaux de Paris ou bien par des chercheurs travaillant en dehors de l'Assistance Publique – Hôpitaux de Paris pour des travaux de recherche entrepris dans le cadre de programmes de recherche.

Nous considérons disposer des informations qui nous permettent de décider.

☐ Nous donnons notre accord pour que les prélèvements biologiques de notre enfant soient utilisés à des fins de recherche.

☐ Nous nous opposons à ce que les prélèvements biologiques effectués sur notre enfant soient utilisés à des fins de recherche.

Nous savons que notre décision n'est pas irrévocable et que nous pouvons donc à tout moment revenir sur notre décision et retirer ou donner notre consentement.

Nous avons également été informé(e) du fait que les données médicales anonymisées concernant notre enfant seront transmises aux chercheurs qui travaillent sur les prélèvements biologiques.
Ces informations sont confidentielles et ne seront partagées qu'au sein du corps médical.
Sur le principe, nous ne nous opposons pas à leur traitement.

TITULAIRES DE L'EXERCICE DE L'AUTORITE PARENTALE
ENFANT APTE A EXPRIMER SA VOLONTE

Fait à :...

Le .... /............./ ....

Nom, prénom : ....
Signature

Nom, prénom : :..
Signature

Nom, prénom de l'enfant : ....
Signature de l'enfant

MEDECIN

Fait à :...

Le .... /............./ ....

Nom, prénom : ....

Signature et tampon du service :
